# Study on the optimization of the deposition rate of planetary GaN-MOCVD films based on CFD simulation and the corresponding surface model

**DOI:** 10.1098/rsos.171757

**Published:** 2018-02-14

**Authors:** Jian Li, Ze-yuan Fei, Yi-feng Xu, Jie Wang, Bing-feng Fan, Xue-jin Ma, Gang Wang

**Affiliations:** 1 School of Electronics and Information Technology, Sun Yat-sen University, Guangzhou 51000, People's Republic of China; 2 Institute of Advanced Technology, Sun Yat-sen University, Guangzhou 510275, People's Republic of China; 3 State Key Laboratory of Optoelectronic Materials and Technologies, Guangzhou 510275, People's Republic of China

**Keywords:** gallium nitride, metal-organic chemical vapour deposition, numerical simulation, epitaxial growth, deposition rate, response surface methodology method

## Abstract

Metal-organic chemical vapour deposition (MOCVD) is a key technique for fabricating GaN thin film structures for light-emitting and semiconductor laser diodes. Film uniformity is an important index to measure equipment performance and chip processes. This paper introduces a method to improve the quality of thin films by optimizing the rotation speed of different substrates of a model consisting of a planetary with seven 6-inch wafers for the planetary GaN-MOCVD. A numerical solution to the transient state at low pressure is obtained using computational fluid dynamics. To evaluate the role of the different zone speeds on the growth uniformity, single factor analysis is introduced. The results show that the growth rate and uniformity are strongly related to the rotational speed. Next, a response surface model was constructed by using the variables and the corresponding simulation results. The optimized combination of the matching of different speeds is also proposed as a useful reference for applications in industry, obtained by a response surface model and genetic algorithm with a balance between the growth rate and the growth uniformity. This method can save time, and the optimization can obtain the most uniform and highest thin film quality.

## 1. Introduction

GaN-based III-V compound semiconductor thin films are important third-generation semiconductor materials, which are widely used in the manufacture of blue-violet light-emitting diodes, semiconductor lasers and high-frequency, high-power electronic equipment [1–6]. Metal-organic chemical vapour deposition (MOCVD) is an important technology for growing GaN thin films due to its advantages such as the large epitaxy area, good reproducibility and high deposition rate [7–11].

Film thickness uniformity is an important index to measure the quality of film growth. In general, the more uniform the film thickness, the more uniform the distribution of components, and the higher the quality of the thin film. The quality of thin film is related to many parameters, such as the gas inlet flow rate, reactor pressure, rotation speed of the base and temperature of the substrate. In order to obtain uniform composition and film thickness, gas flows are required to be laminar. In such a laminar flow, vortices do not occur near the substrate, the gas flow in the direction parallel to the substrate has a uniform temperature field in the direction vertical to the substrate with a high temperature difference, and the particle concentration near the substrate should be as uniform as possible [12–16]. In general, coefficient variation of less than 5% is considered to be important for usable thin films. However, owing to the actual flow state in the reaction chamber, the temperature field and chemical composition of the reaction chamber cannot be visualized by the equipment operator, which introduces unknowns in the process of growth and adjustment.

Fortunately, computational fluid dynamics (CFD) is highly useful for the development of MOCVD equipment. Dimitrios *et al*., in an early study of the cavity flow state [17–19], investigated the particle flow of TiO in a vertical spray reaction chamber, and using the fluid model calculation results and particles in the reaction chamber of the control flow graph experiment, obtained the flow state to determine the feasibility of the numerical simulation. In addition, Theodoropoulos and others investigated the chemical mechanism model [20,21] to study the reaction chamber and coupled field, and proposed a model for the influence of different process parameters on the deposition rate under different conditions. With the improvement of GaN-MOCVD growth mechanism models, scholars such as Mitrovic studied the influence of different process parameters on deposition rate and optimized the design of reaction chambers [22–31].

At present, the planetary MOCVD chamber can adequately solve the problem relating to the thickness uniformity of large volume and large capacity film deposition. Planetary MOCVD is conducive to large size, large modulus and production of thin films, because it can be operated at low rotational speeds; this avoids large centrifugal forces that lead to film delamination. However, there is little research on the influence of the coupling between different rotation speeds on the flow rate and deposition rate. However, a few research groups have theoretically studied the method to adjust the speed of the different regions to find the optimum deposition rate. It is a difficult problem to adjust the uniformity of the film because of the complex coupling field. The simplest method is to adjust film deposition rate by rotating speed.

This study uses the rotation speeds of the substrate and wafers as variable parameters, and other conditions remain unchanged. The quality of MOCVD films was simulated and optimized, and the variation coefficient of thin film thicknesses was used as a measure of the uniformity and the quality of thin film growth. We present the rotation speeds of substrate and wafers as the input variables, the deposition rates as outputs, and the variation coefficient of the deposition rates are used as a response. First, 100 sets of input and output values are obtained by the design of experiment (DOE) method, and then the response surface model is constructed; finally, optimal results are obtained by using a response surface model (RSM) and a genetic algorithm.

## 2. Mathematical formulation

### 2.1. Governing equations

GaN film growth occurs in a state of low pressure, laminar flow and steady state during the growth of MOCVD. Its mathematical model includes the conservation of mass, momentum, energy and individual species.

The conservation equations and conservation equation of momentum are provided [32–34]:

2.1 ∇⋅(ρv)=0

and

2.2 $$\nabla \cdot (\rho v\cdot v)=\nabla \bar{\bar{\tau }}-\nabla p+\rho g,$$

where *ρ* and ***v*** are the density and velocity vectors of the gas mixture, respectively, and *p* and ***g*** are the pressure and gravitational acceleration, respectively. $\bar{\bar{\tau }}$ is the shear stress tensor expressed by

2.3 $$\bar{\bar{\tau }}=\mu (\nabla v+{\left(\nabla v\right)}^{T})-\frac{2}{3}\mu (\nabla v)\cdot I,$$

where *µ* is the viscosity, and *I* is the unit tensor.

The energy equation is given by

2.4 $$C_{p}\nabla \cdot (\rho vT)=\nabla \cdot (k\nabla T)+\overset{N}{\underset{i=1}{\sum }}\frac{H_{i}}{M_{i}}\nabla \cdot J_{i}-\overset{N}{\underset{i=1}{\sum }}\frac{H_{i,0}}{M_{i}}R_{i},$$

where *C*_p_ is the specific heat capacity at a constant pressure, *k* is the thermal conductivity and *T* is the temperature. In addition, $H_{i},\;\;H_{i,0},\;M_{i}$ and ***J_i_*** represent the molar enthalpy, enthalpy of formation, molar mass and diffusion flux of the species, respectively.

We employ a finite rate translation model to simulate the growth of GaN, which can be described by

2.5 $$\nabla \cdot (\rho vw_{i})=-\nabla \cdot J_{i}+M_{i}\overset{K}{\underset{j=1}{\sum }}R_{j}^{i},$$

where *w_i_* is the mass fraction of species *i* and $R_{j}^{i}$ is the net volumetric rate of creation of species *i* during reaction *j*.

The density of the gas mixture is defined by

2.6 $$\rho =\frac{P_{\text{op}}M_{w}}{RT},$$

where *P*_op_, *M*_w_ and *R* are the operating pressure, molecular weight of the gas and universal gas constant, respectively.

### 2.2. Reaction mechanisms

At present, the typical GaN-MOCVD is carried by the carrier gas ammonia (NH_3_), carrying the metal-organic source precursor Ga(CH_3_)_3_ (TMG) and nitride through the top of the uniquely designed spray mixing device into the reaction chamber. From detailed studies by other researchers, it is known that the complex gas will undergo complex gas and surface chemical reactions in the MOCVD reaction chamber and produce a large number of intermediate products. This paper uses the existing GaN growth reaction mechanism, including five gas-phase reactions and five surface reactions to simulate the growth [20,21,31,35], as shown in tables 1 and 2, respectively.

**Table 1.** Gas-phase kinetic scheme for MOCVD of GaN from TMG and ammonia. *k*_0_: activation energy (kcal/mol), *E*: pre-exponential factor (1/s), *β*: temperature influence factor.

reactions	*k*_0_	*E*	*β*
$(\text{G}1)\;\text{Ga(C}{\text{H}}_{3}{\text{)}}_{3}\to \text{Ga(C}{\text{H}}_{3}{\text{)}}_{2}+\text{C}{\text{H}}_{3}$	$3.5\times 10^{15}$	59.5	0
$(\text{G}2)\;\text{Ga(C}{\text{H}}_{3}{\text{)}}_{2}\to \text{GaC}{\text{H}}_{3}+\text{C}{\text{H}}_{3}$	$8.7\times 10^{7}$	35.4	0
$(\text{G}3)\;\text{Ga}{\left({\text{CH}}_{3}\right)}_{3}+\text{N}{\text{H}}_{3}\to {\left(\text{C}{\text{H}}_{3}\right)}_{3}\text{Ga}:\text{N}{\text{H}}_{3}$	$1\times 10^{12}$	0	0
$(\text{G}4)\;\text{(C}{\text{H}}_{3}{\text{)}}_{3}\text{Ga}:\text{N}{\text{H}}_{3}\to \text{Ga(C}{\text{H}}_{3}{\text{)}}_{3}+\text{N}{\text{H}}_{3}$	$1\times 10^{14}$	18.5	0
$(\text{G}5)\;{\left(\text{C}{\text{H}}_{3}\right)}_{3}\text{Ga}:\text{N}{\text{H}}_{3}\to {\left(\text{C}{\text{H}}_{3}\right)}_{2}\text{Ga}:\text{N}{\text{H}}_{2}+\text{C}{\text{H}}_{4}$	$1\times 10^{14}$	49	0

**Table 2.** Gas–surface interactions in metal-organic vapour phase epitaxy (MOVPE) of GaN. S denotes a free surface site and *s* = 1 denotes a unity sticking coefficient at zero coverage.

reactions	*k*_0_	*E*
$(\text{S}1)\;\text{Ga(C}{\text{H}}_{3}{\text{)}}_{3}+\text{S}\to \text{Ga(B) }+3\text{C}{\text{H}}_{3}$	*s* = 1	0
$(\text{S}2)\;\text{Ga(C}{\text{H}}_{3}{\text{)}}_{2}+\text{S}\to \text{Ga(B) }+2\text{C}{\text{H}}_{3}$	*s* = 1	0
$(\text{S}3)\;\text{GaC}{\text{H}}_{3}+\text{S}\to \text{Ga(B) }+\text{C}{\text{H}}_{3}$	*s* = 1	0
$(\text{S}4)\;\text{(C}{\text{H}}_{3}{\text{)}}_{3}\text{Ga}:\text{N}{\text{H}}_{3}+2\text{S}\to \text{GaN(B) }+3\text{C}{\text{H}}_{4}$	*s* = 1	0
$(\text{S}5)\;{\left(\text{C}{\text{H}}_{3}\right)}_{2}\text{Ga}:\text{N}{\text{H}}_{2}+2\text{S}\to \text{GaN(B) }+2\text{C}{\text{H}}_{4}$	*s* = 1	0

To evaluate the uniformity of film thickness, the coefficient of variation is introduced. It is an absolute value reflecting the degree of data dispersion, and measures the degree of variation of the observed data.

2.7 $$\begin{array}{r} \bar{x}=\frac{\sum_{i=1}^{n}x_{i}\times S_{i}}{S} \end{array}$$

2.8 $$\begin{array}{r} C=\frac{\sqrt{1\text{/}n-1\sum_{i=1}^{n}{\left(x_{i}-\bar{x}\right)}^{2}}}{\bar{x}}, \end{array}$$

where $\bar{x}$ and *x_i_* are the average deposition rate and the growth rate of the *i*th location of the area, respectively, *S_i_* and *S* are the area of the *i*th location and the area of the zones between *a* and *b*, respectively and *C* is the coefficient of variation.

## 3. Physical model

### 3.1. Geometry description

**Figure 1.**
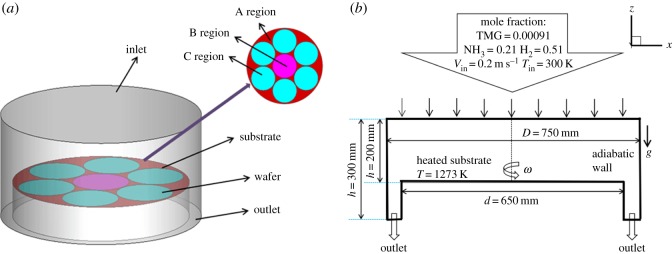
(*a*) GaN-MOCVD Reactor model. (*b*) Gas flow diagram of GaN-MOCVD.

The simulation was carried out in an independently developed conceptual reactor, containing seven 6-inch wafers for GaN-MOCVD, as shown in figure 1. The carrier gas consists of a mixture of N_2_ and H_2_. The group III source element, Ga, and the group V source, N, are uniformly sprayed into the reactor through vertical pipes. After the gas mixture flows through the heated substrate, these two components chemically react to produce monomer molecules, which collide, condense, crystallize and grow into a molecular cloud of particles to eventually form a heterostructured thin film on the substrate surface. The molecular cloud that is not deposited on the substrate leaves the reactor with the unused reactants, by-products and carrier gas through the exhaust flow channel on the sidewall.

The turntable is rotated in three areas, with A being the rotation speed of the substrate, B the central wafer rotation speed and C the rotation speed of the surrounding six wafers, as labelled in figure 1*a*. In order to ensure uniform deposition rate, the substrate and the seven wafers rotate at different speeds. The deposition rate is affected by many factors, such as the gas inlet velocity, substrate rotation speed and pressure in the reactor. In addition, the substrate is large, which creates difficulties in achieving uniform film thickness. Therefore, 12 points are selected on the substrate to study the speed regularity in different regions of the turntable. Average deposition rate along the circles is adopted to measure the thin film thickness.

### 3.2. Boundary conditions

**Figure 2.**
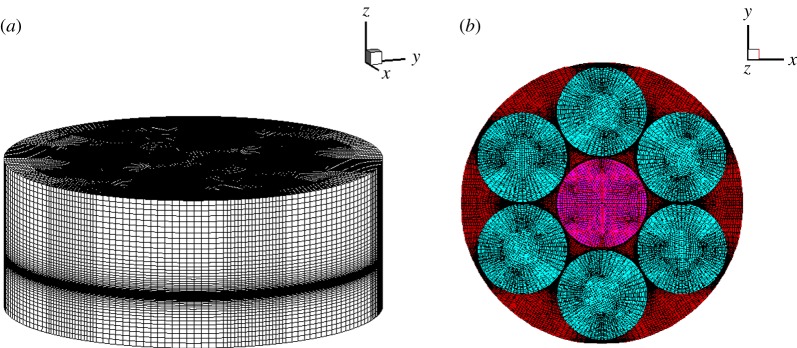
(*a*) The complete mesh of the GaN-MOCVD reactor. (*b*) Substrate grid diagram of GaN-MOCVD reactor.

As shown in figure 2, a hexahedral mesh and local refinement are used in areas where the flow rate and temperature gradient are large. We ensure numerical accuracy of the flow field in the reactor by maintaining the same mesh density in the intersection. Before we start the experiment, a grid sensitivity analysis is performed for the MOCVD reactor model to check its grid independency.

The uniformity of film thickness is not a dimensioned value, and it can be indicated by the coefficient of variation. Generally, the smaller the coefficient of variation, the smaller the degree of numerical dispersion, and the more uniform the film thickness.

The system was modelled using the Fluent (v. 14.0) fluid dynamics software. The convergence criteria were that the residual value of the energy is less than 10^−6^, and the other variables are less than 10^−4^.

The following boundary conditions were imposed:

(1) The growth of the semiconductor material is a continuous transient-state process under the control of mass transmission.
(2) The simulation parameters are provided in table 3 and the boundary conditions are set on the basis that the flow rate of the carrier gas, operating pressure, temperature and rotation speed of the substrate are changed within a certain range.
(3) Laminar flow is adopted because the Reynolds number is less than 2300.
(4) The graphite plate has good thermal conductivity and the temperature difference within the susceptor is very small, considering that the temperature is constant.
(5) A pressure-outlet boundary condition is adopted such that the given outlet static pressure is 0 Pa.

**Table 3.** Simulation parameters.

susceptor temperature (K)	1273	gas inlet temperature (K)	300	A rotation speed (r.p.m.)	20
gas velocity (m s^−1^)	0.2	outer wall temperature (K)	300	B rotation speed (r.p.m.)	5
reactor pressure (torr)	12.5	mole percentage of NH_3_ (%)	0.21	C rotation speed (r.p.m.) (relative to the B region)	20
mole percentage of TMG (%)	0.00091	mole percentage of H_2_ (%)	0.51		

## 4. Results and discussion

### 4.1 Simulation results under the reference conditions

**Figure 3.**
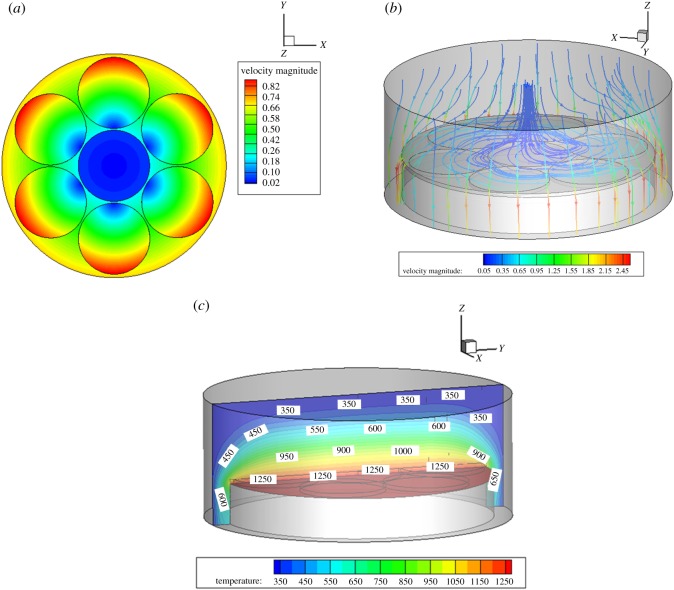
(*a*) Substrate rotation speed in different regions. (*b*) Velocity streamline diagram in MOCVD. (*c*) Temperature profile of reaction chamber.

The results in figure 3 present the flow field, temperature field and deposition rate of the MOCVD reaction chamber under the conditions given in table 1. Figure 3*a* shows the velocity profiles of three regions at different rotation speeds. The flow velocity at the inlet, thermal buoyancy and the flow field of each turntable of the substrate are shown in figure 3*b*. It can also be seen that the inner ring of the entrance acts to encourage gas deposition over the substrate, and the outer ring acts to stabilize the flow field. Figure 3*c* shows the temperature contours in the reaction chamber. The temperature boundary layer, which has a large temperature gradient, is within 5 mm of the substrate surface. The temperature distribution outside the boundary layer is relatively uniform, in the range of 1100–1273 K, which is suitable for the growth and deposition of thin films.

**Figure 4.**
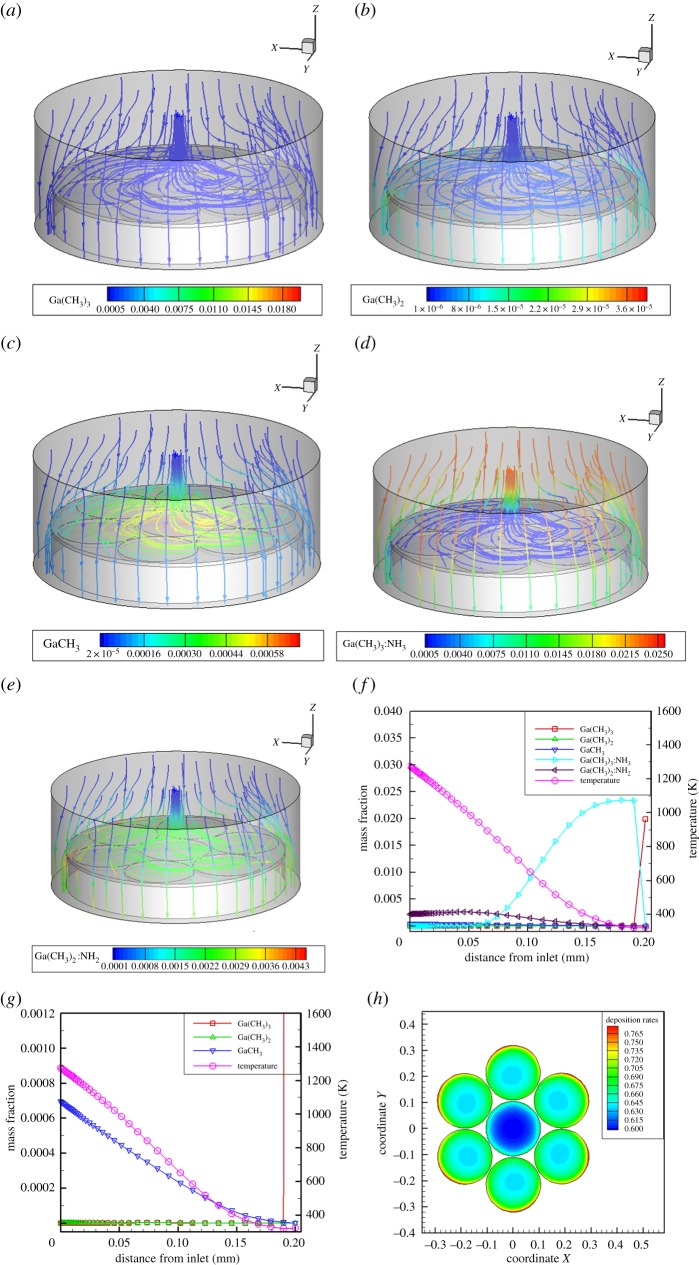
Mass fractions of Ga-containing species under reference conditions in MOCVD. (*a*) Ga(CH_3_)_3_, (*b*) Ga(CH_3_)_2_, (*c*) GaCH_3_, (*d*) Ga(CH_3_)_3_:NH_3_, (*e*) Ga(CH_3_)_2_:NH_2_. Mass fractions of Ga-containing species along the symmetry axis of MOCVD under reference conditions; (*f*) all species; (*g*) low-concentration species. (*h*) Film deposition rate.

Figure 4*a–e* is the mass fraction cloud chart distribution of each Ga component in the reaction chamber under the reference conditions. Figure 4*f,g* shows the mass fraction distribution of each component along the central axis of the reactor. Combined with the two diagram observations, it is known that in the upper part of the substrate, because reaction G3 does not need activation energy, Ga(CH_3_)_3_ and NH_3_ will quickly form adducts (G3) in the reaction chamber. The adduct is filled in the range of distance entrance 1 mm. When the adduct is near the high-temperature boundary layer, Ga(CH_3_)_3_:NH_3_ decomposes backward to generate Ga(CH_3_)_3_ and NH_3_ (G4), and the maximum concentration is generated at a distance of 5 mm from the inlet, where the temperature is about 400 K. However, the undecomposed Ga(CH_3_)_3_:NH_3_ is diffused to the high-temperature area and removed CH_4_ to produce an amino group Ga(CH3)_2_:NH_2_ (G5). The maximum concentration occurred at the distance entrance 12.5 mm, where the temperature is about 900 K. The amino compounds continue to deposit here to produce GaN films. At the same time, when the temperature rises to the activation energy barrier, which can be crossed over in reactions G1 and G2, Ga(CH_3_)_3_ will be further decomposed, and Ga(CH_3_)_2_ and GaCH_3_ will be generated near the substrate. However, the Ga(CH_3_)_3_ concentration of the product of direct decomposition of GaCH_3_ is at least two orders of magnitude lower than the product of its addition reaction, Ga(CH3)_2_:NH_2_. The deposition rate of the substrate film is shown in figure 4*h*; however, it can be seen that the deposition rate is very uneven, with a film thickness variation coefficient of 5.94%. Therefore, we studied the speed of each region in order to obtain a more uniform deposition rate.

### 4.2. Influence of rotational speed on the flow field and temperature field

**Figure 5.**
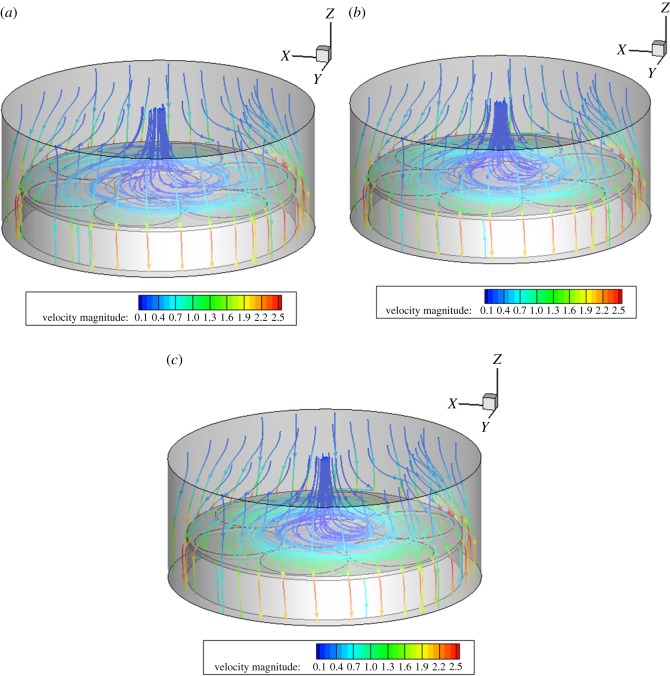
Velocity streamline diagram at different speeds in region A. (*a*) 30, (*b*) 40 and (*c*) 50 r.p.m.

When other benchmark conditions are unchanged, and when the rotation speeds of region A are 30, 40 and 50 r.p.m., the rotation speeds of different bases have little influence on the flow field of the reaction chamber. In the intersection area of the turntables A and C, the streamline becomes more fluent with the increase in the speed, and the flow-field distribution on the base still keeps more uniform and straight, as shown in figure 5.

**Figure 6.**
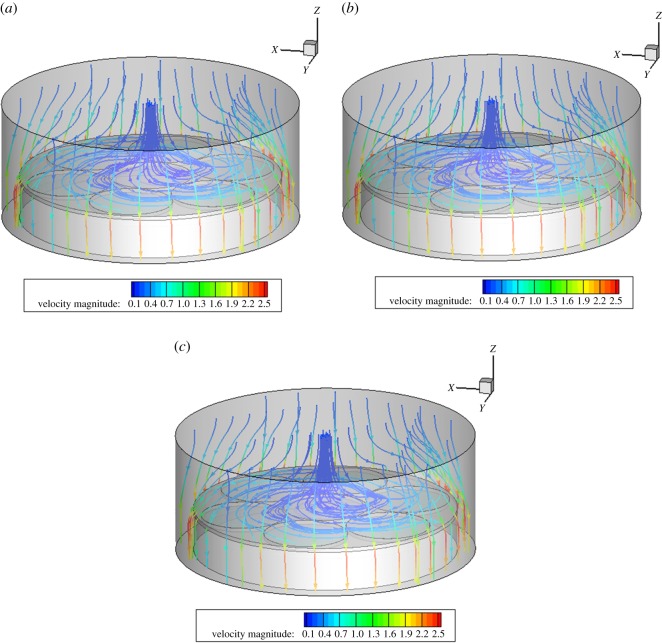
Velocity streamline diagram at different speeds in region B. (*a*) 10, (*b*) 15 and (*c*) 20 r.p.m.

When other benchmark conditions are unchanged, and when the rotation speeds of region B are 10, 15 and 20 r.p.m., the rotation speeds of different bases have little influence on the flow field in the reaction chamber. The distribution diagram of the flow field at the three speeds is basically similar, and the distribution of the flow field is even more uniform above the base, as shown in figure 6.

**Figure 7.**
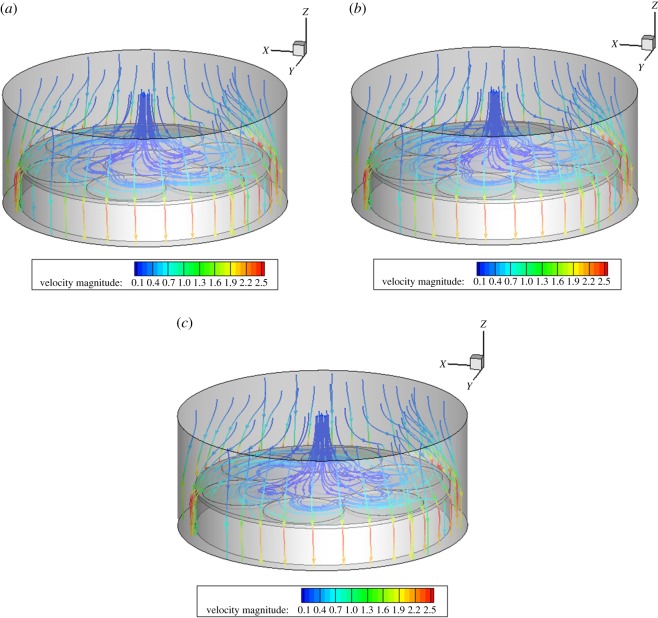
Velocity streamline diagram at different speeds in region C. (*a*) 30, (*b*) 40 and (*c*) 50 r.p.m.

When other benchmark conditions are unchanged, and when the rotation speeds of region C are 30, 40 and 50 r.p.m., the rotation speeds of different bases have certain influence on the flow field of the reaction chamber. Three-speed flow-field distribution is slightly different in the intersection region of substrates A and C with the increase in the rotational speed fluctuations. With the increase of speed and cyclotron eddy above substrate C, it will have a certain impact on the film deposition. Above the base, a relatively uniform distribution flow is maintained, as shown in figure 7.

The different rotational speeds of the substrates of the three regions have little effect on the temperature field of the reaction chamber, which is basically consistent with that shown in figure 3*c*. (For further details, refer to electronic supplementary material, figures S1–S3.) Because the rotation speed does not increase significantly, it does not affect the temperature field in speed range.

### 4.3. Influence of rotational speed on species transport

Figures 8–10 show the mass fraction of the Ga(CH_3_)_2_:NH_2_ components in MOCVD at the three regions at the speed of the region. The change in the substrate speed also does not change the relative size of the material concentration in the boundary layer, and changes only slightly under the influence of the speed. All the components of the three regions at different speeds are shown in the support file electronic supplementary material, figures S4–S15.

**Figure 8.**
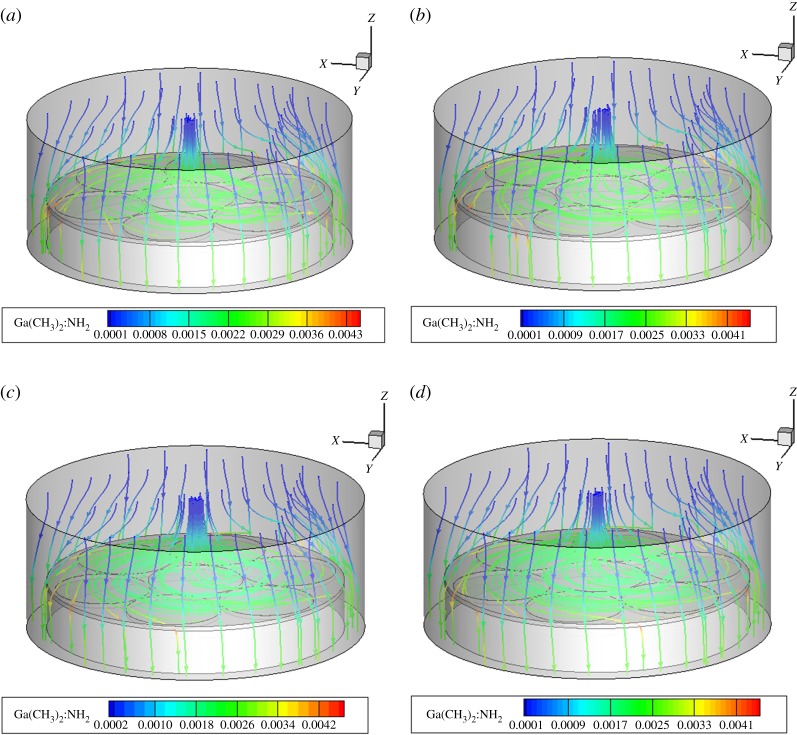
Mass fractions of Ga(CH_3_)_2_:NH_2_ of MOCVD under the rotation conditions of region A. (*a*) 20, (*b*) 30, (*c*) 40 and (*d*) 50 r.p.m.

**Figure 9.**
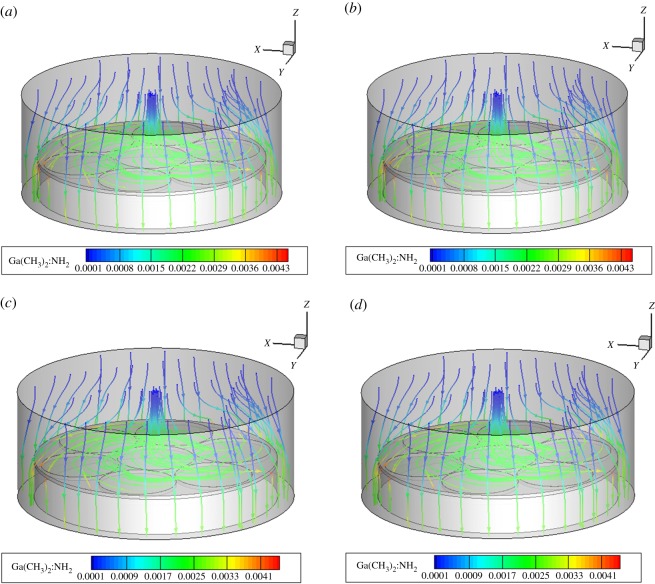
Mass fractions of Ga(CH_3_)_2_:NH_2_ of MOCVD under the rotation conditions of region B. (*a*) 5, (*b*) 10, (*c*) 15 and (*d*) 20 r.p.m.

**Figure 10.**
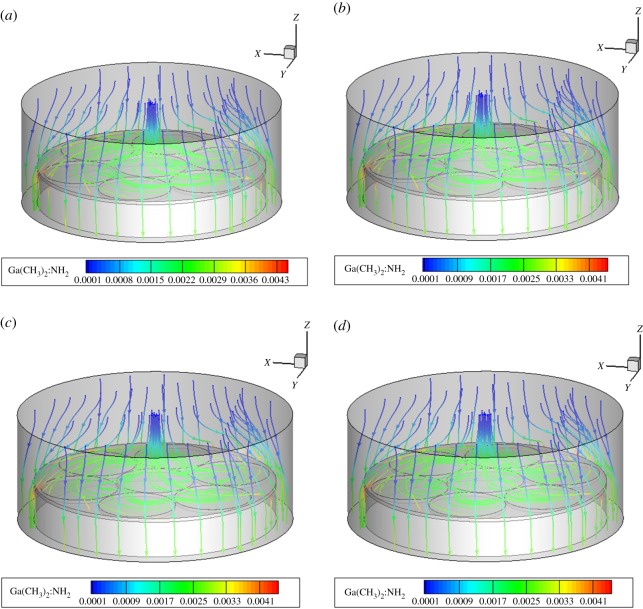
Mass fractions of Ga(CH_3_)_2_:NH_2_ of MOCVD under the rotation conditions of region C. (*a*) 20, (*b*) 30, (*c*) 40 and (*d*) 50 r.p.m.

In the three regions and at different rotational speeds, the concentration distribution of substances in the concentration boundary layer is Ga(CH_3_)_2_:NH_2_, GaCH_3_ and Ga(CH_3_)_2_, and Ga(CH_3_)_3_ and Ga(CH_3_)_3_:NH_3_ disappear in the cavity. That is, the speed of changing the substrates of different regions does not change the rule that the Ga(CH_3_)_3_ and the NH_3_ plus-pyrolysis paths dominate. The main growth precursors of the film growth are still Ga(CH_3_)_2_:NH_2_ and GaCH_3_.

In summary, the characteristics of planetary MOCVD involve material deposition at low rotational speeds. Therefore, changing the substrate speed in a relatively low range will not change the overall temperature field, flow field and component distribution. It will also not affect the dominance of addition and pyrolysis paths in MOCVD. It only affects the concentration change of the Ga gas component near the substrate rotation region, which further affects the change in the film deposition rate.

### 4.4. Influence of rotational speed on deposition rate

**Figure 11.**
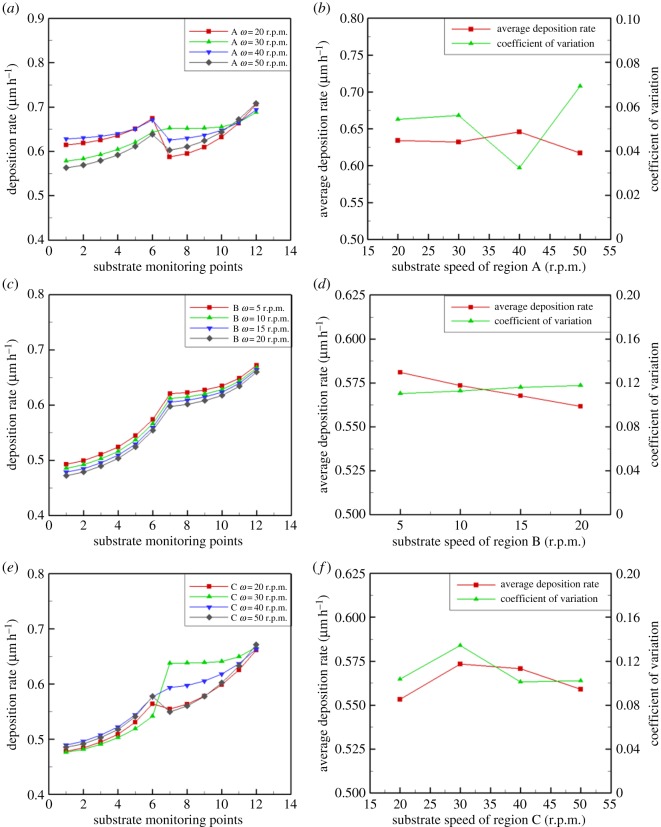
Effect of region A rotation speed on (*a*) deposition rate and (*b*) coefficient of variation. Effect of region B rotation speed on (*c*) deposition rate and (*d*) coefficient of variation. Effect of region C rotation speed on (*e*) deposition rate and (*f*) coefficient of variation.

In the case of other conditions being unchanged, the deposition rate changed as the rotation speeds increase, as shown in figure 11. Figure 11*a*,*b* shows the deposition rate dependence on the rotation speed of region A. As the speed increases, the growth rate increases slightly, while the growth uniformity becomes slightly variable. This shows that the effect of substrate rotation speed on deposition rate and composition distribution is very small. Figure 11*c*,*d* shows the deposition rate dependence on the rotation speed of region B. The surrounding wafers are symmetrically distributed and rotate at the same speed, and the upper gas flow includes the central wafer to drive the uniform spread of the air distribution. As the speed increases, the growth rate and the growth uniformity again increase by a small amount. Figure 11*e*,*f* shows the deposition rate dependence on the rotation speed of region C. Rotation of the central wafer causes central cavity flow; with increasing speed, both the growth rate and the growth uniformity increase first, and then decrease. The central cavity flow not only causes the gas flow to spread around the substrate but also promotes an overall uniform airflow. It shows that the influence of central wafer rotation speed on the deposition rate distribution is relatively large. Figure 11 shows that in the intersection of the inner ring and the outer circle, due to the mismatch of the speed and the flow field, the deposition rate of the connecting parts in the two regions is significantly changed.

## 5. MOCVD process parameters optimization

### 5.1. The optimization goal and optimization scheme

Rotation speeds of the substrate, central wafer and surrounding wafers are *X* = {*x*_1_, *x*_2_, *x*_3_}, and the average deposition rates of 12 circles are *Y* = {*y*_1_, *y*_2_, *y*_3_, *y*_4_, *y*_5_, *y*_6_, *y*_7_, *y*_8_, *y*_9_, *y*_10_, *y*_11_, *y*_12_}.

We propose that the problem of film thickness uniformity control is converted to the question: how to adjust the rotation speeds *X* to minimize the coefficient of variation of film uniformity.

5.1 $$\text{Min }C(Y):\;Y=f(X)\;5\leq x_{1}\leq 20;\;20\leq x_{2}\leq 50;\;20\leq x_{3}\leq 50.$$

where, *x_i_* is the different zone speed, *f*(*X*) is the function between the inlet velocity and the average deposition rate, and *C*(*Y*) is the function of film thickness variation coefficient.

In order to obtain the most uniform film thickness, the variation coefficient of the 12 average deposition rates must be minimized. If the other conditions remain unchanged, the optimal result is obtained by adjusting the values of A, B and C, as shown in table 4.

**Table 4.** MOCVD process parameters.

variables (r.p.m.)	A	B	C
lower bound (r.p.m.)	20	5	20
upper bound (r.p.m.)	50	20	50
output	12 average deposition rates		

Numerical simulation takes a lot of time; thus, complete simulation using all the possible range of values of rotation speed will be a very large and time-consuming project. Therefore, in this study an RSM method was used. One hundred sets of data were simulated by adjusting the values of A, B and C, and then these 100 sets of inputs (A, B and C) and outputs (12 deposition rates) were used to construct the input and output mapping relationship. Then 10 sets of new data were randomly selected to verify the accuracy of the model. Finally, a genetic algorithm was used to optimize the response surface model to find the most uniform (minimum coefficient of variation) result.

### 5.2. Construction and analysis of RSM model

**Figure 12.**
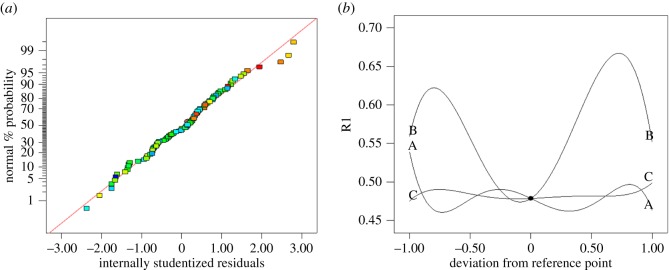
(*a*) Normal probability plot of residuals. (*b*) Influence of speed in different regions.

The model has 12 deposition rate results; however, we can select just one of the results of the analysis due to the other results being similar. Figure 12*a* shows the normal probability plot of residuals, in which most of the points are in the normal distribution, and the fitting results of the model are very good. Figure 12*b* shows the effect of a single variable on the outcome: B value is the most influential, followed by A, with C having the least impact.

The substrate rotation speed, A, has a small numerical change range, and is acting on the gas flow in the whole reactor, so that the change is relatively stable. The rotation speed of the central wafer, B, causes a vortex generated by rotation to spread throughout the reaction chamber, so that the local vortex drives the airflow distribution in the whole chamber to drive the reactant diffusion around, and thus has the greatest impact on deposition rate. The rotation speed, C, of the six circularly distributed wafers, and the induced vortices are symmetrical, resulting in the least impact. Thus, the influence of different rotating speeds on the flow field leads to the change of deposition rate of substrate film.

**Figure 13.**
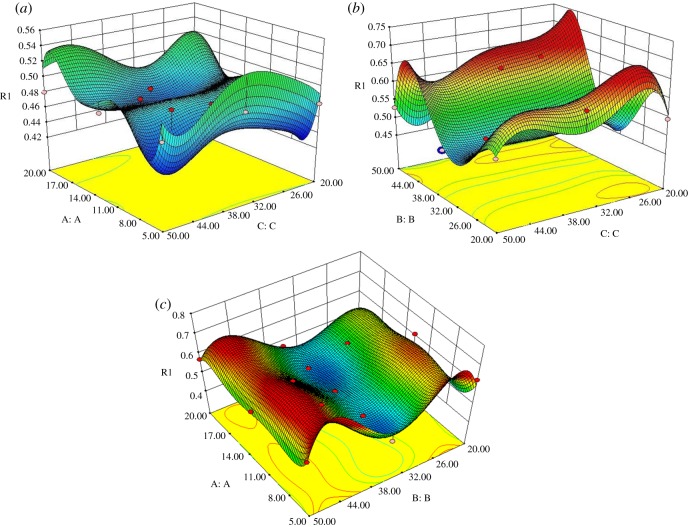
Three-dimensional RSM diagram of the influence of different regions on substrate deposition rate. (*a*) A and C coupled effect. (*b*) B and C coupled effect. (*c*) A and B coupled effect.

Figure 13 shows the three-dimensional response surface model diagram, which illustrates the effect of the interaction of two parameters on the result. It not only shows the single factor effect, which is included in figure 12*b*, but also shows the interaction of two factors of A, B and C. From a single factor A surface alone, as the rotation speed increases, the fluctuation trends of A and figure 12*b* are in agreement; the same is true for factors B and C. The surface showing the A and C coupled effect is not stable, leading to the difference in deposition rate. However, the surfaces of B and C are coupled, and the A and B coupled effect is relatively stable.

### 5.3. Predication performance of RSM model and optimization result

Eight sets of new data were randomly selected, as shown in the electronic supplementary material. The RSM-predicted results were compared with the CFD simulation results and the average deposition rate error is no more than 5%. Therefore, the RSM-predicted and simulated values are consistent in both the numerical values and the changing trends, and for the subsequent optimization of the coefficient of variation, it is highly dependent on the accuracy of trend. Therefore, the RSM model can be used as the corresponding model for the input and output of the MOCVD reactor, which is used for the following optimization process.

**Figure 14.**
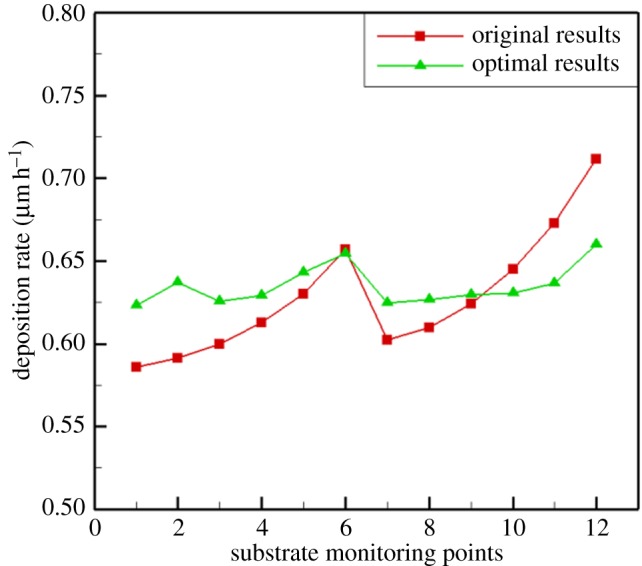
Deposition rate distribution of the optimized substrate.

Based on the corresponding relation of the input and output, the genetic algorithm is used to find the parameter value of the minimum coefficient of variation of the 12 deposition rates. The optimization results are shown in figure 14. The optimal rotation speeds are A = 40 r.p.m., B = 18 r.p.m. and C = 24 r.p.m., which provide a variation coefficient of 1.87%.

## 6. Conclusion

In this study, the MOCVD planetary and wafer rotation speeds were optimized for uniform film thickness, by both numerical simulation and using an RSM model. The important conclusions from this study are as follows.

(1) When the inlet flow rate, turntable temperature and internal pressure are stable, the influence of different speed regions on the temperature field is not obvious, but there is a certain disturbance in the speed and species, further affecting the deposition rate. Especially with the increase in the speed of region C, the disturbance is more obvious.
(2) The accuracy of the RSM model is high, such that the predicted value and simulation value can be used to map the relationship between inputs and outputs, and it can be used to optimize the process parameters. The optimum rotation speed of different regions is obtained by using the optimized model, which provides a theoretical basis for obtaining the best film deposition rate. The coefficient of variation of film thickness across the entire substrate is reduced from 5.00 to 1.87%.
(3) A model with a large number of grids is used to construct the RSM model, which can investigate the influence of each process parameter on the result, significantly reducing simulation time and cost.

## Data accessibility

Data accompanying this paper can be found at Dryad (http://dx.doi.org/10.5061/dryad.63821) [36].
